# Precursory worldwide signatures of earthquake occurrences on Swarm satellite data

**DOI:** 10.1038/s41598-019-56599-1

**Published:** 2019-12-30

**Authors:** A. De Santis, D. Marchetti, F. J. Pavón-Carrasco, G. Cianchini, L. Perrone, C. Abbattista, L. Alfonsi, L. Amoruso, S. A. Campuzano, M. Carbone, C. Cesaroni, G. De Franceschi, Anna De Santis, R. Di Giovambattista, A. Ippolito, A. Piscini, D. Sabbagh, M. Soldani, F. Santoro, L. Spogli, R. Haagmans

**Affiliations:** 1 Istituto Nazionale di Geofisica e Vulcanologia, Via di Vigna Murata 605, Roma, 00143 Italy; 2 Now at School of Remote Sensing and Geomatics Engineering NUIST, Nanjing University of Information Science and Technology, Nanjing, China; 3 Now at Univ. Complutense de Madrid, Facultad CC. Físicas, Avd. Complutense, s/n – Madrid 28040, Spain & Geoscience Institute IGEO (CSIC – UCM), Madrid, 28040 Spain; 4 Planetek Italia srl, via Massaua 12, Bari, 70132 Italy; 5 Now at ASI, Via del Politecnico snc, Roma, 00133 Italy; 6 SpacEarth Technology, Via di Vigna Murata 605, Roma, 00143 Italy; 7 European Space Agency, ESTEC, Keplerlaan 1, NL-2201 AZ Noordwijk, The Netherlands

**Keywords:** Natural hazards, Geomagnetism, Seismology

## Abstract

The study of the preparation phase of large earthquakes is essential to understand the physical processes involved, and potentially useful also to develop a future reliable short-term warning system. Here we analyse electron density and magnetic field data measured by *Swarm* three-satellite constellation for 4.7 years, to look for possible *in-situ* ionospheric precursors of large earthquakes to study the interactions between the lithosphere and the above atmosphere and ionosphere, in what is called the Lithosphere-Atmosphere-Ionosphere Coupling (LAIC). We define these anomalies statistically in the whole space-time interval of interest and use a Worldwide Statistical Correlation (WSC) analysis through a superposed epoch approach to study the possible relation with the earthquakes. We find some clear concentrations of electron density and magnetic anomalies from more than two months to some days before the earthquake occurrences. Such anomaly clustering is, in general, statistically significant with respect to homogeneous random simulations, supporting a LAIC during the preparation phase of earthquakes. By investigating different earthquake magnitude ranges, not only do we confirm the well-known Rikitake empirical law between ionospheric anomaly precursor time and earthquake magnitude, but we also give more reliability to the seismic source origin for many of the identified anomalies.

## Introduction

A large earthquake comes after a long-term preparation phase composed of different stages of seismicity evolution driven by the continuous but variable tectonic stress^1,2^. The understanding of the underlying physical processes is likely to deliver the most reliable prediction method^3^. As it is practically impossible to follow this process at the level of the fault (typically at least tens of km depth), an alternative is to study if the lithosphere interacts with the above atmosphere and ionosphere, i.e. assuming a Lithosphere-Atmosphere-Ionosphere Coupling (LAIC^4–8^), during this long-term phase, but with particular attention to the very last stages. Co-seismic coupling in the atmosphere is well established^9^, while the possible pre-earthquake coupling is more debated. A recent example is the possible ionospheric electron density enhancement before large earthquakes^10^.

To explain the LAIC effects, different models have been proposed in the last years. Freund^5^ proposed a mechanism based on the theory of *p-holes* (positive holes), which are produced by the stress along the fault. When p-holes reach the Earth’s surface, they could ionize the atmosphere. These charged particles could create instability in the mesosphere and on the edge of the ionosphere. The mechanisms were tested successfully in laboratory^11^. An alternative mechanism is proposed by Pulinets and Ouzounov^6^, based on gas and fluid that could rise up toward the surface in the preparatory phase of the earthquake. Another model was provided by Kuo *et al*.^12^, which relies on a numerical simulation. It takes into consideration the role of the Earth’s magnetic field, suggesting a possible mechanism of alteration of the ionosphere that improves their previous model^13^.

A third possible mechanism for the pre-earthquake electric field appearance is the possibility of modification of the electric field around the height of 100 km due to internal atmospheric gravity waves^14^.

De Santis *et al*.^7^, Pulinets and Boyarchuk^15^ and Hayakawa^16^ presented a general review about the processes that can occur in the atmosphere and ionosphere before and during an intense earthquake, and their possible correlations.

To detect the pre-earthquake ionospheric anomalies, various parameters can be monitored by ground-based equipment such as ionosondes^17–23^, Global Navigation Satellite System (GNSS) receivers^24,25^ and ULF magnetic field sensors^26–28^.

Parrot^29^ reviewed the most important results from the space investigations. The seminal satellite mission DEMETER^30^ was specifically designed to possibly identify a wide range of electromagnetic pre-earthquake signals^31^ and its statistical analyses were encouraging, pointing to LAIC above some reasonable level of randomness for 6.5 years of earthquakes^32–34^.

A promising application of the geomagnetic field monitoring by the *Swarm* satellite mission to the 2015 Nepal earthquake (M7.8) showed a correlation between the magnetic anomalies and earthquakes temporal pattern^35^. A similar approach, applied to other earthquakes provided promising results^36–41^.

Here we investigate the correlation of *in-situ* electron density and magnetic field anomalies from *Swarm* satellites with earthquakes. For this scope, a Worldwide Statistical Correlation (WSC) analysis based on a superposed epoch approach has been applied to *Swarm* data for the first time. This approach is applied in a time window around earthquakes that occurred during a period of four years and eight months since 1 January 2014.

## Results

### Worldwide statistical correlation analysis

We analyse the electron density (Ne) and magnetic field data from the three *Swarm* satellites to detect possible anomalies associated with the earthquakes from 1 January 2014 to 31 August 2018 (30 August for Ne). For the magnetic field anomalies we consider only the Y-East magnetic field component in the analysis (see Methods section for more details).

The worldwide earthquake data were extracted from the USGS (United States Geological Survey) catalogue (https://earthquake.usgs.gov), in terms of the time of occurrence, hypocenter location (geographical coordinates and depth) and magnitude. We selected the same time span of the satellite data (from 1 January 2014 to 31 August 2018) and declustered the catalogue to remove dependent earthquakes^42^, in order to avoid bias in superposed epoch approach. For the purpose of this study, only earthquakes with the hypocentral depth less than 50 km are considered, since deeper earthquakes are less likely to affect the ionosphere^43^. The final dataset of 1312 M5.5+ worldwide shallow earthquakes was the base for the statistical correlation analysis with *Swarm* satellite data (see Methods section for more details).

**Figure 1:**
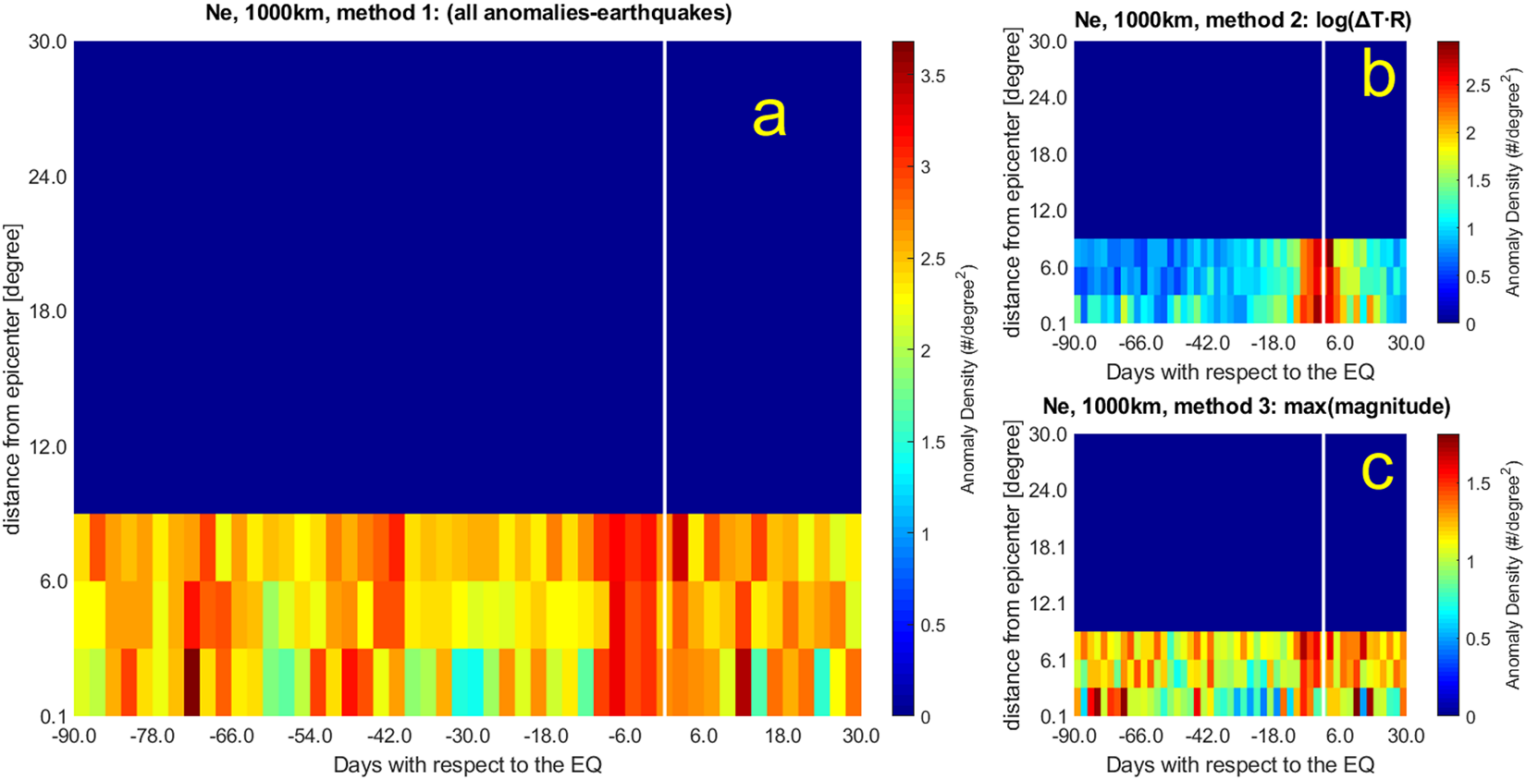
Worldwide Statistical Correlation (WSC) in terms of a superposed epoch approach applied to electron density Ne with threshold k_t_ = 3.0 considering a distance of 1000 km from earthquake (EQ) epicentre; *x*-axis presents the days before (negative days) or after (positive days) the EQ occurrence, while *y*-axis shows the distance from the earthquake epicentres in degrees. The analysis has been made for all hours (H24). (**a**) No constraints are imposed on anomaly-EQ association (Method 1). (**b**) Association EQ-Anomaly is made minimizing the value of Log_10_(ΔT∙R), where ΔT is the precursor time and R the distance of the anomaly with respect to EQ epicentre (Method 2). (**c**) The same as (**b**) but assigning just the EQ with maximum magnitude for each anomaly (i.e. Method 3 or MaxM method). Most of the concentrations appear before and around the EQ occurrences. For the meaning of *d* and *n*, please refer to the main text. Please note the vertical extent of the bin is 3°.

**Figure 2:**
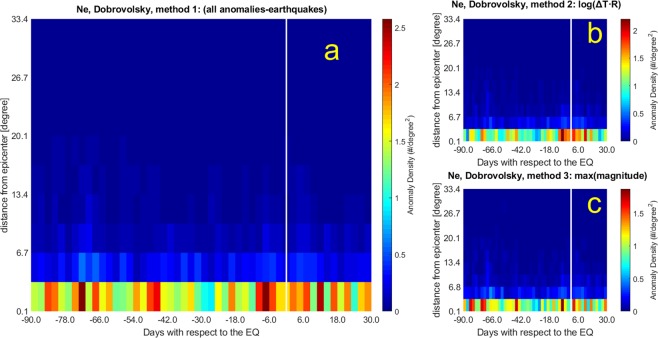
The same as Fig. 1 (Ne WSC analysis) but considering the Dobrovolsky area (DbA). Please note the vertical extent of the bin is 3.4°.

Electron density and Y magnetic component signals from all the *Swarm* satellites have been analysed track by track by a moving window to provide two anomaly datasets according to a universal threshold k_t_, one for each investigated quantity (see Methods section for algorithms description). Then we apply WSC to extract those anomalies (if any) associated in space and in time to the earthquakes and to obtain the superposed epoch diagrams (see Methods section for more details). Figures 1 and 2 show the correlation results when applied to the electron density by considering:

1. Anomaly threshold k_t_ = 3.0 within 1000 km from epicentres (bins of 2.4 days × 3 degrees).

2. Anomaly threshold k_t_ = 3.0 within Dobrovolsky radius^44^ (bins of 2.4 days × 3.34 degrees).

**Figure 3:**
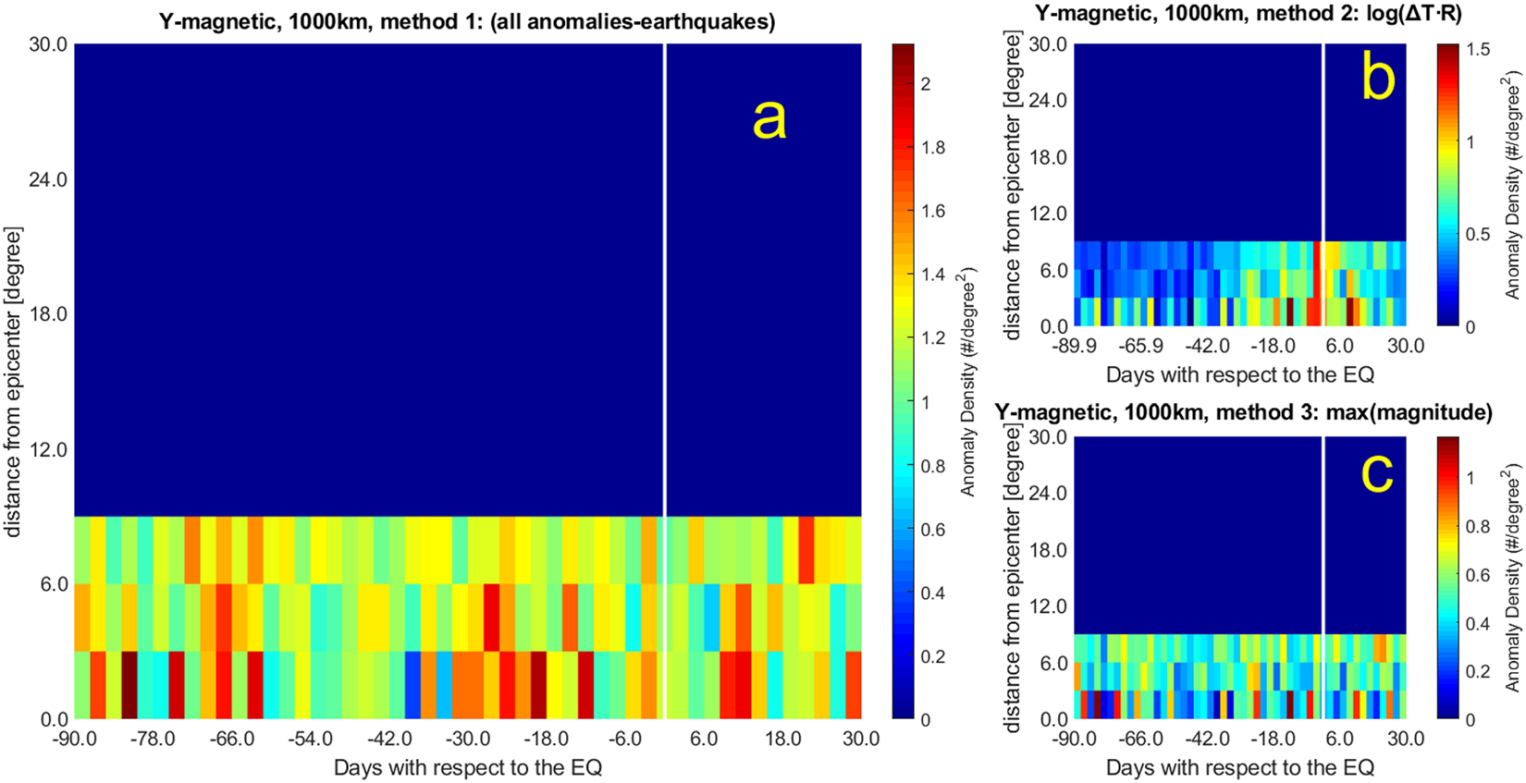
The same as Fig. 1 but for Y magnetic field component and k_t_ = 2.5. (**a**) Method 1; (**b**) Method 2; (**c**) Method 3.

**Figure 4:**
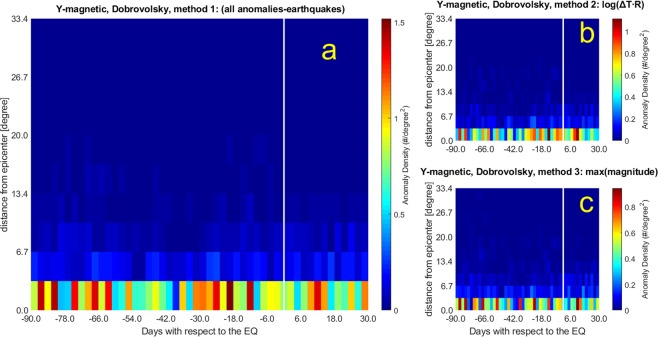
The same as Fig. 3 (Y magnetic field WSC analysis) but considering the Dobrovolsky Area (DbA).

Figures 3 and 4 show the correlation results when applied to the Y magnetic field data by considering:

3. Anomaly threshold kt = 2.5 within 1000 km from epicentres (bins of 2.4 days × 3 degrees).

4. Anomaly threshold kt = 2.5 within Dobrovolsky radius (bins of 2.4 days × 3.34 degrees).

Although our algorithm accepts any value of the threshold k_t_, we applied a larger value (k_t_ = 3.0) for Ne than Y (k_t_ = 2.5), because the former quantity is more variable than the latter, producing usually more outliers.

Please note that Figs. 1 and 3 concern with analyses up to 1000 km from epicentres, however the diagrams extend the representation even after 1000 km (this explains the abrupt passage from colors to blue at 9°), although they do not have any physical meaning (in these cases, there are no anomaly data detected after 1000 km), only to maintain simple comparison with those of Figs. 2 and 4 that extend up to around 30°. From Figs. 1 to 4, the panels (a) include all possible anomaly-earthquake associations, i.e. anytime an anomaly falls inside the area and the time span investigated around the event a point is inserted. The advantage of this method (here also called Method 1) is to not make any hypothesis, but we know that it is extremely unlikely that an anomaly could be produced by two or even more seismic events at the same time and this case could appear too frequently when this method is applied. To overcome this unlikely situation, we propose also two other methods that are both plausible but introduce other assumptions: the first one (here also called Method 2) is shown in panels (b) where we associate the anomaly to the space-time closer earthquake by minimizing Log_10_ (ΔT∙R), being ΔT the time between anomaly occurrence and earthquake origin time, while R is the distance between the location of the anomaly and the epicentre. The second one (here also called Method 3) is shown in panels (c) and associates the anomaly to the largest magnitude event among all earthquakes compatible with that anomaly.

By looking at Figs. 1 and 2, large precursory concentrations of Ne anomalies fall few days (6–8 days) before the earthquakes, although some meaningful concentrations are also noticeable about 45 days and between 70 and 85 days before the events. We estimated the statistical significance of the correlation by means of two statistical parameters that indicate how much the maximum concentration is higher than a typical random maximum concentration (*d* parameter) and how many standard deviations σ the concentration is far from the simulated ones (*n* parameter) (for more details see the Methods). The statistical significance of the electron density anomalies is very low for the 1000 km analyses (*d* = 1.0–1.3 and *n* = 0.3–4.1), being comparable with typical random distributions of anomalies when Method 1 and 2 are applied, while is high for Dobrovolsky Area (DbA) analyses (*d* = 1.5–1.7 and *n* = 4.6–15.1). The 6–8 days anomaly concentration confirms previous results from DEMETER satellite^34^. The other longer precursory times had never been investigated so far and are the topic of the next section, where we will explain and justify this aspect in terms of the Rikitake empirical law^45^. It is encouraging that the methods that introduce some earthquake physical model and parameters such as Dobrovolsky strain radius as a limit of the spatial search (Fig. 2) and their maximum magnitude (Fig. 2c) show a higher statistical significance, giving empirical evidence for the seismic origin of most of these anomalies.

We notice that in the analyses performed within a radius of 1000 km from epicentres (Fig. 1a–c) some unrealistic concentrations (although not the largest ones, which always fall at the closest band to epicentres) appear at farther distances. We can interpret these features as due to anomalies actually belonging to other closer earthquake epicenters, but included in the analyses, especially for smaller earthquake magnitudes. We suspect that this feature contributes to the low statistical significance of these kinds of analyses. However, this effect disappears when analysing the data according to the Dobrovolsky area (Fig. 2a–c), for which the distance of interest for smaller magnitude earthquakes is much smaller than 1000 km.

In Fig. 3b,c, and Fig. 4b the anomalies found in the Y magnetic field component analysis maximize around 12 days before the earthquake occurrences. Figure 3a,c (and Fig. 4a,c) show concentrations at even longer time intervals (about 80 days), the same period that we found for electron density anomalies. Figure 4a presents the largest concentration around 20 days before the earthquakes, a precursor time that appears also in other analyses, although with less significance.

The Y magnetic field component analyses show larger values of *d* and *n* (*d* = 1.4–2.1; *n* = 6.0–16.6) than Ne analyses (*d* = 1.0–1.7; *n* = 0.3–15.1).

The adoption of the Dobrovolsky strain radius slightly affects the main temporal features of the WSC analysis: although the anomaly density decreases (e.g., compare Fig. 2 with Fig. 1), the periods of higher density before earthquakes are confirmed. From the spatial point of view, anomalies cluster closer to the epicentres (i.e. always within the first spatial band).

Table 1 reports the main properties of the analyses performed over the real cases as well the results on their reliability, obtained by comparison with random distributions through the *d* and *n* parameters, whose values range from 1.0 to 2.1 and from 0.3 to 16.6, respectively. Bold numbers in *d* and *n* evidence the best cases for which the real analyses are well distinct from random simulations (selected as *d* ≥ 1.5, because the density is equal to or larger than 50% of random distribution, or *n* ≥ 4, because the probability to be random is equal to or less than 0.1%). Generally the *d* values increase when the Dobrovolsky area and the maximum earthquake magnitude criteria are considered (this is consistent with the expectation that larger earthquakes cause greater anomalies). The best results are reported when correlation is applied to the Y component of the magnetic field. Regarding the largest anomaly concentrations, some appear in almost all analyses, in particular 7, 12, 20 and 82 days before the earthquakes. Having enlarged the temporal window of analysis with respect to previous studies allowed us to detect also high precursor times such as 86 and 82 days before the earthquakes. They look significant and appear both in Ne and in Y.

**Table 1:** Statistics for the real cases analysed in the paper in all space-time interval compared with the values of the random data analyses (Table S1).

	Figure	Anomalies in the 120 day window	EQs with anomalies	Day of largest concentration	Anomalies in the max	EQs in the max	*[Dmax/D*_0_]_*real*_	*d*	*n*
Ne: k_t_ = 3.0 1000 km Method 1	1a	32568	1170	−72, −7	126	40	1.1	1.0	1.7
Ne: k_t_ = 3.0 1000 km, Method 2 log(ΔT∙R)	1b	14846	992	+2*, −2	93	35	1.0	1.0	0.3
Ne: k_t_ = 3.0 1000 km, Method 3 MaxM	1c	14846	888	−82, −72, −7*, +12*, +14*	63	23	0.7	1.3	**4.1**
Ne: k_t_ = 3.0 DbA Method 1	2a	7731	722	−72, −7	90	33	1.3	**1.5**	**8.7**
Ne: k_t_ = 3.0 DbA, Method 2 Log(ΔT∙R)	2b	5958	611	−7	77	33	1.3	**1.5**	**4.6**
Ne: k_t_ = 3.0 DbA, Method 3 MaxM	2c	5958	495	−7	65	27	1.3	**1.7**	**15.1**
YMag: k_t_ = 2.5, 1000 km Method 1	3a	15747	1171	−82, −19, −12, + 12	71	27	1.8	1.4	**7.5**
YMag: k_t_ = 2.5, 1000 km, Method 2 Log(ΔT∙R)	3b	6605	857	−12, + 10	53	19	1.8	**1.6**	**6.0**
YMag: k_t_ = 2.5, 1000 km, Method 3 MaxM	3c	6605	751	−82, −12	40	14	1.5	**2.0**	**10.3**
YMag: k_t_ = 2.5, DbA, Method 1	4a	3987	538	−19	53	24	2.8	**2.0**	**16.6**
YMag: k_t_ = 2.5, DbA, Method 2 Log(ΔT∙R)	4b	2805	437	−12	39	12	2.5	**1.8**	**9.5**
YMag: k_t_ = 2.5, DbA, Method 3 MaxM	4c	2805	328	−86, −82, −24, +12	33	15	2.8	**2.1**	**15.8**

Ne/YMag at the lefter column indicate Ne/YMag real analysis. Day(s) of the largest concentration(s) (usually Brown colors; sometimes also Red in Figs. 1–4) of anomalies is(are) taken with respect to the earthquake (EQ) occurrence, where negative means before and positive means after it, with ±1.2 day uncertainty. For the analyses of Ne the anomalies in the whole space-time window were 58692, while for the Y magnetic field were 22142. For convenience to the reader, there is also the column of the corresponding Figure to which the results refer.

Please note that *d* is actually estimated as the ratio of the [D_max_/D_0_]_real_ with that [D_max_/D_0_]_rand_ obtained from the mean of 100 simulations with the same exact number of anomalies, as shown in Table S1. *n* measures the significance of the [D_max_/D_0_]_real_ with [D_max_/D_0_]_rand_ as times of standard deviations (see main text). Bold numbers are those of the real cases that are significantly different than the random simulations in terms of *d* ≥ 1.5 or *n* ≥ 4. Four cases present both values as significant: all Y magnetic field component analyses in the DbA, and the Y magnetic field component analysis in 1000 km with MaxM, i.e. Method 3.

*At the third and farthest band from epicentres.

In general, one method to select the association of the anomaly with the earthquake emphasises anomalies closer to earthquake occurrences (Log(ΔT∙R), panel b), while another one tends to show also longer precursory times (earthquake magnitude, panel c). Applying all methods, however, we find signatures in electron density and Y magnetic field component anticipating the earthquakes, from a few days to around 80 days and the statistical correlation obtained makes this precursory feature compelling.

### Rikitake law and its interpretation as result of a diffusion process

From what we carried out and summarized in Table 1, earthquake precursors would appear at different lead times, i.e. we would expect not only a single concentration but several concentrations at different times in advance before the earthquake occurrences, according to the range of earthquake magnitudes. A formula relating precursor time ΔT in days to magnitude M was proposed by Rikitake^45^: Log_10_(ΔT) = *a* + *b*M. To deeper investigate this concept, we extended our analysis to 500 days before the earthquake occurrences, applying Method 1, i.e. without imposing any earthquake-anomaly association, in order to not favour closer or farther anomalies with respect to earthquake occurrences. We considered both the electron density and the Y-component of the magnetic field within the Dobrovolsky area and grouped the correlations for the following individual bands of earthquake magnitude: 5.5–5.9, 6.0–6.4, 6.5–6.9, 7–7.4, 7.5+. Of course, the choice of 500 days could be critical because for a large number of events (i.e. the ones occurred in the first 500 days of 4.7 years of investigated period) not all the time domain was covered by our analysis. On the other hand, this would allow us to look at longer potential precursor times.

**Figure 5:**
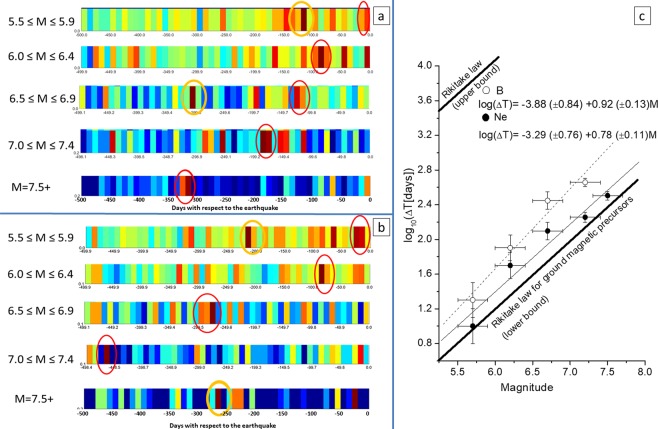
Worldwide Statistical Correlation (WSC) in terms of a superposed epoch approach applied to electron density with threshold k_t_ = 3.0 (**a**) or Y magnetic field with threshold k_t_ = 2.5 (**b**) considering the DbA around the EQ epicentre and different ranges of magnitude values. Please note that the investigated time interval is 500 days (versus 90 days before and 30 days after in the previous analyses) before the EQ occurrences and each temporal bin is of 10 days (versus 2.4 days of previous analyses). Only the closest spatial band to the EQ epicentre is shown; for convenience colour palettes are not shown, although blue stands for the lowest density (close to zero) and brown for the largest density (that differs from case to case). Red ovals indicate the larger concentrations considered for the Rikitake law; yellow ovals are not taken into account for the reason given in the main text. (**c**) shows the Rikitake law for electron density Ne (black circles and thin fitting solid line) and Y magnetic field (empty circles and thin fitting dash line). Also the corresponding *a* and *b* coefficients are given, together with the upper and lower bounds of the Rikitake law for ground magnetic precursors^45^.

Figures 5a,b show the WSC results, for Ne and Y component of the magnetic field, respectively (now every single bin is 10 days × 3.34 degrees large). At a first glance, the identification of the largest group of anomalies (red ovals), taken as considering two adjacent bins of these new diagrams, is straightforward in all magnitude intervals with a few exceptions (yellow ovals). Among them, however, some can be easily excluded: the yellow ovals in the middle panel of Fig. 5a and in the top panel of Fig. 5b are actually two-bin combinations less significant than the corresponding red ovals. In addition, for the top panel of Fig. 5a, we exclude the yellow oval because is too distant from earthquake occurrence, giving more credit to the closer concentration (red oval). The results for M7.5+ are not sufficiently robust, because the number of earthquakes is rather small (16) in the 4.7 years of the study. However, to test the Rikitake law^45^, we included this largest range of magnitude for the electron density. We fit the same Rikitake functional law to our precursor times with respect to the central value of magnitude for each band. We estimated the error bars for the magnitude as the half width of the investigated range and for the time interval as the bin span of the anomalies concentration. Figure 5c shows the results for both Ne (black circles and thin fitting solid line) and Y (empty circles and thin fitting dash line), together with lower and upper bounds of the Rikitake law for magnetic ground precursors^45^ (thick lower and upper lines). It is surprising that, within the estimated errors, we find similar *a*,*b* values (*a* = −3.29 ± 0.76, *b* = 0.78 ± 0.11 for Ne and *a* = −3.88 ± 0.84, *b* = 0.92 ± 0.13 for Y) to those proposed for ground magnetic observations by Rikitake^45^ (see Methods section).

Apparently the two fitting lines for Ne and Y are distinct, but both fits are within the errors. In addition, for both quantities, it appears that earthquake precursors occur within a day for events with magnitude below 4.2, although is it very unlikely that these weaker earthquakes could produce an effect in the ionosphere^20^.

On the other hand, the greater the earthquake magnitude, the greater the difference between the ΔT referred to Ne and Y-component: for instance, for M7.5, the Ne relation provides ΔT = 363 days, while Y relation gives ΔT around 1000 days. This could explain why we do not find a statistical significance in the M7.5+ analysis for Y magnetic component.

This result strongly supports the empirical law found by Rikitake^45^ for ground magnetic observations, even extended it to electron density and magnetic field satellite data, with a little adjustment of the coefficient values.

The Rikitake law is reasonable for the process of earthquake generation and coupling with the above atmosphere and ionosphere layers with simple arguments (more details are shown in Methods). Assuming a lithospheric process of stress diffusion^46^ across the Dobrovolsky strain radius R_Db_^44^, we obtain the relationships *a* = −Log($4\pi {\rm{D}}\,$) and *b* = 2β, *D* is the coefficient of diffusion and β is the Dobrovolsky exponent: 0.43 (see Methods for all the passages). We can verify them by comparing the *b* value obtained from Rikitake (around 0.8) that, within the error, resembles the value of 0.86 deduced from Eq. 7b in Methods. From the value of *a*, we can even estimate *D*. Although *a* value has large uncertainty, i.e. *a* ≅ −2 (Rikitake), *a* ≅ −3.3 and *a* ≅ −3.9 (in our analysis of Ne and Y, respectively), we can take the central value of *a* ≅ −3, obtaining *D* ≅ 100 m^2^/s, which is one-two order more than a reasonable value for the crust^1^. However, it is really interesting that this same order of diffusivity can be found for slow earthquakes when a diffusion model is considered^47^. This interesting coincidence would merit more future attention, potentially opening new perspectives in seismological studies.

## Conclusions

The electron density and magnetic field WSC analyses applied to about 4.7 years of *Swarm* satellite observations highlight that anomalies appear to occur before the earthquake occurrences, between a few days and 80 days before the earthquakes, with larger peaks at around 10, 20 and 80 days. We find that in all analysed cases considering the DbA, the largest concentration of pre-earthquake anomalies is statistically significant by a factor up to around 2 (i.e. d ∼ 2) times the simulated data, with up to 15–17 σ (i.e. n = 15–17). We note that the detected anomalies seem better correlated with earthquakes of stronger magnitude.

We confirm linear relation between the LogΔT and magnitude, and the found *a*, *b* coefficients are, within the estimated errors, close to those proposed by Rikitake^45^. We also provide a simple explanation of this relationship and show that the empirical expressions we found for satellite data anomalies are consistent with a stress diffusion process in the crust as that producing slow earthquakes.

The Rikitake law, confirmed by the separate analyses of Fig. 5a,b at different ranges of magnitude, supports the argument that the precursor times are related to the earthquake magnitude. Its expected effect would be to theoretically smear out eventual peaks in the analyses of all magnitude earthquakes, what we do not actually find in Figs. 1–4. However, our results are not in contradiction for two main reasons: i) The analysis limited to 90 days before and 30 days after earthquake occurrence is heavily influenced by the preponderance of low magnitude earthquakes, so concentrations are more confined within the closest times before earthquakes, while the longer time precursors, likely produced by larger earthquakes, are out of the analysed temporal interval. ii) The analyses reported in Fig. 5 are extended to 500 days before, to include longer precursor times, typical of larger magnitude earthquakes tend to appear well in advance with respect to earthquake occurrences. However, the law is an empirical law which is not “exclusive”, so an earthquake could even provide other precursors in different time.

Although this investigation would support the LAIC with clear statistical significance, another clear message emerges: not all earthquakes are in the favourable conditions to produce significant effects in the ionosphere. Only a portion of them (we estimate something around 40%; see section of Methods) generates a non-negligible electromagnetic effect that cannot be due to simple chance. Several causes can be attributed to this deficiency: insufficient satellite passes available, an inappropriate coversphere (e.g. vegetation), adverse meteorological conditions, and/or still not perfect anomaly detection strategy.

More detailed analyses, as the study of the type of earthquakes analysed, according to their occurrence in subduction zones or along strike-slip faults, in the land or under the sea, in interplate or intraplate, and the inspection of single anomalies, would help to better understand the physics behind the possible coupling phenomena. In this respect, a prolongation of the *Swarm* satellite mission is greatly encouraged: for example, with at least 10 years of data, longer precursor times could be investigated without loss of statistical significance. Data from other quasi-polar satellites, equipped with magnetometers and Langmuir probes (e.g. the Chinese seismo-electromagnetic satellite^48^), would also be useful for this scope.

A final point is clearly important here: since the ionospheric anomalies are causally related to what happens inside the Earth and at the ground-to-air interface, the corresponding parameters should be, or even must be, included in any further study. This is implicit in the Geosystemics concept^8^, by which we can consider the Earth system as an ensemble of cross-interacting parts. Therefore, it is mandatory to consider a multiparametric point of view to address this kind of complex phenomena, by combining seismological, atmospheric and ionospheric information^8,40^.

## Methods

### Satellite data

*Swarm* mission is an ESA satellite mission^49^ composed of three identical quasi-polar orbiting satellites, two (Alpha and Charlie satellites, also named as *Swarm*-A and *Swarm*-C, respectively) at lower orbit (around 460 km above the Earth’s surface) and the third (Bravo or *Swarm*-B) at the highest orbit (around 510 km). The satellites were placed in orbit on 22 November 2013 and are still orbiting around the Earth. The original configuration with *Swarm*-A and *Swarm*-B flying in parallel and *Swarm*-C in a higher orbit^49^ was changed to the present one because of early problems (from 5 November 2014) in the scalar magnetometer of *Swarm*-C.

The satellite payloads comprise, among others, a Langmuir probe (LP) and two magnetometers, i.e. a vector fluxgate magnetometer (VFM) and an absolute scalar magnetometer (ASM). These sensors have been here analysed systematically to detect possible pre-earthquake Lithosphere-Atmosphere-Ionosphere Coupling (LAIC) electron density and magnetic field anomalies from space.

The Ne data used in this work are measured by the LP of *Swarm* satellites with a sampling rate of 2 Hz. The input data have been provided by the original *Swarm* Advanced product called “2_Hz_Langmuir_Probe_Extended_Dataset”. This dataset is provided in Common Data Format (CDF) and freely available in the ESA *Swarm* FTP and HTTP Server swarm-diss.eo.esa.int (the *Swarm* data are also available from VIRES web platform: http://vires.services). The current release of 2 Hz Langmuir Probe Extended Dataset (Release 101) is the ESA reprocessing of all *Swarm* Electric Field Instrument data from the beginning of the mission to 30 August 2018.

According to ESA, the current values of electron density are up to a few 10% too high at low density (https://earth.esa.int/web/guest/missions/esa-eo-missions/swarm/data-handbook/preliminary-level-1b-plasma-dataset). This is not an issue for the purposes of our anomaly detection, as it is not based on the absolute value itself, but its derivative as reported later in the text.

We also considered scalar intensity (F) and vector X,Y,Z magnetic components. The data are at 1 Hz sampling that correspond to the low resolution magnetic data contained in the Level 1b (L1b) products. All the L1b magnetic data are provided by ESA in Common Data Format (CDF) and hosted in the ESA *Swarm* server.

In order to select data without evident troubles/problems during the satellite flying (https://earth.esa.int/web/guest/swarm/data-access/quality-of-swarm-l1b-l2cat2-products), we took into account the quality flags associated with both the electron density and magnetic field data.

In detail, we extracted different information from original CDF files, including the type of satellite, i.e. A for Alpha, B for Bravo, and C per Charlie, the UTC time, data quality flags, the electron density or the vector magnetic components in NEC (North, East, Centre) reference frame by the VFM instrument and the magnetic absolute intensity by ASM instrument (for A, B and C satellites; however, as ASM of C after 5 Nov. 2014 is out of work, the total intensity is calculated from the Cartesian magnetic components given by VFM instruments).

The error of the magnetic field measured by *Swarm* satellites can be estimated to be less than 0.3 nT, with a typical value of 0.1 nT^50^.

### Algorithms for electromagnetic anomaly detection

The approach to identify anomalies is based on two novel algorithms, i.e. *NeAD (Electron Density Anomaly Detection)* and *MASS* (*MAgnetic Swarm anomaly detection by Spline analysis*), applied to the electron density Ne (2 Hz sampling) and magnetic field (components and total intensity-1Hz sampling) from *Swarm* satellites (A,B,C), respectively. These algorithms share the main features that we highlight below.

For each day and for all the satellites tracks (the daily number of semi-orbit for each satellite is about 32), the Local Time (LT) and the geomagnetic latitudes are evaluated, the latter based on a tilted geocentric dipole field from the last generation of the IGRF (International Geomagnetic Reference Field) global model of the geomagnetic field (IGRF-12^51^). Then, the first time derivatives of Ne and of the magnetic field are estimated as the first difference values divided by the time interval between two consecutive samples. Finally, a fit with cubic splines is applied to remove the long term trend.

Figures S1, S2 show an example of the output from *NeAD* and *MASS*, respectively, carried out for two different epochs and for *Swarm* C: April 27, 2015 (two days after the M7.8 Nepal earthquake occurred on 25 April 2015) and February 14, 2016 (almost two weeks before the M7.8 Sumatra earthquake occurred on 2 March, 2016). The track number, descending (D)/ascending (U), the corresponding local time (LT) and UTC, the geospace conditions given by the Dst and a_p_ geomagnetic indices, areprovided. The geographical area of interest with satellite track (red), the Dobrovolsky area (yellow oval; circular on the terrestrial sphere) and the earthquake epicentre (green star) are also shown. It is worth noticing that, from Figure S2, an anomalous signal is visible especially in the East magnetic component (Y), while the total intensity does not show appreciable variability^39^. This could be simply explained by field aligned current processes that do not practically affect the total intensity of the magnetic field vector, but only its direction^35^. Accordingly, instead of analysing the whole magnetic field, we focused only on the magnetic Y component because it is less affected by external perturbations than X and Z components^52^ and this increases the possibility to detect Earth internal source anomalies.

To detect anomalies of interest for the WSC analysis, *NeAD* and *MASS* outputs (for the entire *Swarm* data set) are further analysed by overlapping sliding windows within ±50° geomagnetic latitude, to limit the effects due to the high latitude ionosphere, and under quiet magnetic conditions in terms of Dst and a_p_ indices (|Dst| ≤ 20 nT, a_p_ ≤ 10 nT) to limit the effects of perturbations coming from the outer space.

We consider overlapping sliding windows of 7.0° latitude length, moving by 1.4° (1/5 of window length) along the whole ±50° geomagnetic latitude range. Since the satellite speed is of about 7.6 km/s, the choice of the 7.0° sliding window allows us to include typical pre-earthquake satellite signals of some tens of seconds into a sufficiently short spatial length^34,53^. The approach with overlapping sliding windows provides an output matrix in which each row identifies a given window and each column contains the following quantities: the date and central time (UTC and LT) of the given window, the satellite (A,B,C), the track number, Dst, a_p_, the root mean square error (rms) over the samples distribution within the given window and the frequency content of the window (the latter not used in this work), together with the root mean square error (RMS) over the whole track in ±50° geomagnetic latitude.

Finally, from the output matrix, we define as anomalous those windows (i.e. the Ne and Y magnetic field component values within them) for which rms > k_t_∙RMS, where k_t_ is an appropriate threshold (normally 2.5–3) and RMS is the root mean square error computed for the whole track.

Summarizing, criteria adopted by *NeAD* and *MASS* to detect anomalies are:

- *Swarm* tracks within ±50° geomagnetic latitude;
- Low magnetic activity: |Dst| ≤ 20 nT, a_p_ ≤ 10 nT during the track acquisition time;
- sliding windows of 7.0° latitude length, moving by 1.4° along the tracks;
- rms (of each sliding window) > k_t_∙RMS (evaluated along the track).

### Worldwide statistical correlation algorithm

The WSC algorithm evaluates the possible correlation in space and time of the detected anomalies by *NeAD* and *MASS* with the earthquake locations and occurrences. The earthquake catalogue was declustered first by extracting all M5+ earthquakes, then detecting and removing the dependent earthquakes, i.e. those earthquakes with magnitude M ≤ M_ms_−1 (M_ms_ is the mainshock magnitude) that happen inside 10 km from the mainshock epicenter and a 10-day time window from its origin time. In the declustered catalogue, the magnitude of the mainshock is replaced by the equivalent magnitude of the seismic cluster. We then selected those shallow earthquakes with M ≥ 5.5 for which LAIC signatures are more likely to be captured as highlighted by Liu *et al*.^20^ who found a dramatic enhancement in the statistical correlation between ionospheric anomalies and earthquakes with magnitude greater than 5.4. In addition, this choice avoids problems of catalogue incompleteness^54^.

WSC cumulates all the anomalies of the same family (Ne or Y magnetic field component) associated with each earthquake (occurring in the time interval normally ranging between 90 days before and 30 days after the earthquakes; but we also extended the time interval up to 500 days before earthquakes to verify Rikitake law; see next dedicated section) in a unique space-time graph by a superposed epoch approach having as common origin the occurrence time and the epicentre of all investigated earthquakes along the available *Swarm* mission data (4.7 years). The horizontal axis of the WSC diagrams is the time lag between the anomalies and the seismic events and the vertical axis is the distance of the anomalies with respect to the epicentre (in degrees). The common time origin is represented by the white vertical line. The colour bar identifies the density level of the anomalies (number of anomalies for squared degree). A simplified flowchart of the WSC algorithm is shown in Figure S3. This method is similar to that one applied to DEMETER satellite data analysis^32–34^, apart from the use of a wider time window around the earthquakes (in Yan *et al*.^34^, for example, it was −15 to 5 days; here we normally considered −90 to 30 days). We considered a longer time window according to the empirical relationship given by Rikitake^45^: for a typical M5.5 earthquake, which is the most frequent in our dataset, we would expect a precursor at a mean advance time of 16 days, in a range from 2 to 144 days (see also Piscini *et al*.^55^). Therefore, extending the analysis to 90 days before the earthquake occurrence, seems to be a good compromise, avoiding border effects in time. In space, we consider either an area comprised by a fixed radius of 1000 km around the earthquake epicentre^34^ or the most popular Dobrovolsky’s circular area (DbA) around the earthquake epicentre, whose radius R_Db_ in km scales with magnitude M, i.e. R_Db_ = 10^0.43M^, as suggested by Dobrovolsky *et al*.^44^. For our selected earthquakes, the radius of this area is between 230 km or 2.2° (M5.5) and 3700 km or 33.4° (M8.3). The DbA is considered a good empirical approximation of the preparation area of an impending earthquake^56^.

In addition to the adopted spatial (anomalies within 1000 km and/or within the DbA from the epicentres) and temporal (anomalies occurring from −90 days to 30 days around the earthquake occurrences) criteria, we present the analysis in three different ways.

The first method (Method 1) does not take into account any assumption, associating each anomaly to all earthquakes that fall inside the analysed space time. In the next two methods, we introduce some limitations to prevent an anomaly from being associated to more than one earthquake, which is very unlikely to happen. This can be done by:

Method 2: Referring a given anomaly to that earthquake for which Log_10_ (ΔT∙R) is minimum, where ΔT is the time lag between the anomaly occurrence and the earthquake (we also call it precursor time), and R is the spatial distance of the anomaly from the earthquake epicentre. This method intends to assign to the anomaly the closest earthquake in space and time. This limitation takes also into account the correlation found between Log_10_ (ΔT∙R) and the earthquake magnitude M when ionosphere anomalies from HF ionosondes are considered^18,19,22,23^.

Method 3: Referring a given anomaly to that earthquake with the greatest magnitude M (also referred as MaxM method), falling inside the analysed space-time window.

The Method 1 has the advantage to not impose any further constraints on the anomalies, but has the disadvantage to have eventually some anomalies with more than one associated earthquake.

We would underline that the selection of a particular method affects only a small percentage of the whole cases. So, all the methods have a common anomaly-earthquake association.

Therefore, we provided space-time distributions, one for each earthquake, of the anomalies (Ne and/or Y-magnetic field component) associated with it. Then we superposed all the distributions imposing a common origin that identifies the earthquake occurrence times and the epicentres. The resulting WSC distribution (one for each parameter, Ne and/or Y magnetic field component) contains all the anomalies overlapped. The density level of the anomalies (number of anomalies per squared degree) is estimated in 50 temporal bins and 10 spatial bins, so each bin of the diagram usually covers 2.4 days (120 days divided by 50 bins). The epicentral distance (vertical extent) of each bin is of 3°, i.e. about 330 km at the Earth’s surface for the 1000 km analyses (or 3.34°, i.e. about 370 km at the Earth’s surface, for the DbA analyses), up to 30° (33.4° for the DbA analyses, corresponding to the largest DbA for the largest magnitude M8.3 in the earthquake dataset). The small difference in the two full-scales is due to the different height of the bins in the two kinds of analysis. In the 1000 km analyses, it allows to complete the whole distance with three full bands, but almost preserving the possibility to compare these analyses with those made across the DbA. In any case, to maintain perfect agreement in all (1000 km and DbA) analyses the anomalies and earthquakes have been counted always in a bin of 3.34°.

The statistical significance of the WSC analysis results is based on the introduction of quality statistical quantities as follows.

To estimate how much reliable is the largest concentration of anomalies, we firstly considered the ratio between the largest concentration of the real anomalies D_MAX_ and that of a theoretical uniform distribution (in space and time) D_0_ of anomalies, i.e. D_MAX_/D_0_.

D_0_ can be analytically defined as:

1 $${D}_{0}=\frac{{N}_{an}}{A\cdot \Delta t}\cdot {N}_{eq}$$

where ${N}_{an}$ is the total number of anomalies in the whole analysed region and in all times; A is the whole area of the analysis in square degrees (in this case it is the area of a tesseral zone between −50° and + 50° of geomagnetic latitude); Δ*t* is the analysed time in days, in this study it is 1703 days, i.e. 4.7 years. *N*_*eq*_ is the number of earthquakes associated with at least one anomaly. Thus, the units of D_0_ are (square degrees × days)^−1^.

D_MAX_ is calculated as:

2 $${D}_{MAX}=\frac{{N}_{exan}}{{A}_{BIN}\cdot \Delta {t}_{BIN}}$$

*N*_*exan*_ is the number of anomalies associated with earthquakes in the bin of the first row (i.e. the anomalies closest to the epicentre) with the largest concentration of anomalies; A_BIN_ is the area of the bin (i.e. the area of the circle or of the annulus region of the first bin around the epicentre); Δ*t*_*BIN*_ is the time width of the bin in the same unit of Δ*t* (2.4 days if we investigate a window from 90 days before to 30 days after the earthquake occurrence).

Note, however, that D_MAX_/D_0_ is biased because the areas associated with the earthquakes could overlap, either in case of considering circles of 1000 km around the epicentres or the effective DbA. In addition, we do not analyse all the temporal periods but only those with low magnetic disturbance (i.e. |Dst| ≤ 20 nT, a_p_ ≤ 10 nT), to roughly “filter out” those anomalies clearly depending on solar geomagnetic activity.

Then, to establish the actual statistical significance of D_MAX_/D_0_, we compare it with its analogous obtained by correlating the real earthquake dataset with a certain number (usually 100) of random distributions of anomalies (with same number of the real cases), almost homogeneous in space and time. The random anomalies are generated assigning to each of them a latitude, a longitude and an occurrence time. The latitude and longitude are selected (with homogeneous probability in space) within the analysed global area (i.e. the area with |geom. latitude| ≤50°). The occurrence time is selected among the geomagnetic quiet times with uniform probability. From the random simulations, we also calculate the average and the standard deviation σ_rand_ of the parameter [D_MAX_/D_0_]_rand_, to be further compared with that one corresponding to [D_MAX_/D_0_]_real_ for the real cases.

Table S1 summarizes the main features of the random simulations, each of them referring to the different criteria adopted in the WSC real cases. For each series of 100 random simulations, we provide also the standard deviation σ_rand_.

Then we consider the statistical parameter *d* defined as the ratio between [D_MAX_/D_0_]_real_ (Table 1), estimated over the real anomaly data, and [D_MAX_/D_0_]_rand_, estimated over a set of simulated random anomaly data (Table S1). That is:

3 $$d=\frac{[{{\rm{D}}}_{{\rm{MAX}}}/{{\rm{D}}}_{0}]{\rm{real}}}{[{{\rm{D}}}_{{\rm{MAX}}}/{{\rm{D}}}_{0}]{\rm{rand}}}\mathrm{.}$$

In this way, *d* would show how much the real maximum concentration is above the expected typical maximum concentration of a random anomaly distribution: the larger is the *d* value, the more the results of the WSC applied to real data deviate from randomness.

Finally, to increase the WSC reliability, we provide also the parameter *n* measuring the significance of the real statistical results with respect to the random distributions, defined as: *n* = ([D_max_/D_0_]_real_ − [D_max_/D_0_]_rand_)/σ _rand_. Also in this case, the larger *n*, the more significant the corresponding real analysis.

### Fraction of earthquakes with ionospheric effects

We can provide a rough estimate of the fraction of earthquakes that produce ionospheric effect in two ways. A way is based on the best cases of Table 1: this estimate is given by the ratio between the earthquakes with anomalies (third column) and the total number of earthquakes, i.e. 1312. Another way is based on the separate analyses of Fig. 5a,b: we can count the number of earthquakes in the largest concentrations that contribute to the Rikitake law. Both estimates point to values between 25% and 55%. Therefore, the given value of 40% is a reasonable estimation.

### Rikitake law

We recall the empirical law by Rikitake^45^, that relates precursor time ΔT with the earthquake magnitude M:

4 $${\mathrm{Log}}_{10}(\Delta {\rm{T}})=a+b{\rm{M}}$$

where *a* = −2.08 (±1.43) and *b* = 0.78 (±0.23), for geomagnetic field ground observations^45^.

Although out of our scope, we sketch some simple reasoning on why the Rikitake law is reasonable for the process of earthquake generation and coupling with the above atmosphere and ionosphere layers. Adopting a lithospheric process of stress diffusion^46^ across the DbA, we can relate the spatial distance R (in km) of the anomaly from the earthquake epicentre with the time ΔT:

5 $$R=\sqrt{4{\rm{\pi }}{\rm{D}}\Delta T}$$

where D is the diffusivity. If we suppose that the first precursor can appear at the beginning of the stress evolution, then ΔT will be the precursor time.

According to Dobrovolsky *et al*.^44^ we can express *R*_*Db*_, as:

6 $${\rm{L}}{\rm{o}}{\rm{g}}{R}_{Db}=\beta M$$

with β = 0.43 and *M* the earthquake magnitude.

If we assume R = *R*_*Db*_, we can replace *R*_*Db*_ in (6) with R of the Eq. (5), so that the coefficients in Eq. (4) become:

7a $$a=-\,\mathrm{Log}(4{\rm{\pi }}{\rm{D}})$$

and

7b $$b=2{\rm{\beta }}$$

From our results we could even deduce D from (7a) and verify the relationship (7b) (see Section before Conclusions).

### Dedication

We would like to make a special dedication to the memory of Eigil Friis-Christensen (1944–2018), lead investigator of the *Swarm* satellite mission. Without him probably nothing of all published works about *Swarm* mission could ever have been written.

## Supplementary information

Supplementary information.
