# Supplementary material for: Precursory worldwide signatures of earthquake occurrences on Swarm satellite data
Source: Sci Rep. 2019 Dec 30;9:20287. doi: 10.1038/s41598-019-56599-1 (PMC6937265; doi:10.1038/s41598-019-56599-1)
Supplement: Supplementary file 1 — Supplementary information. [file 41598_2019_56599_MOESM1_ESM.pdf]

Supplementary Information

Article in *Scientific Reports*

Precursory worldwide signatures of earthquake occurrences on Swarm satellite data

A. De Santis[1*], D. Marchetti[1,2], F.J. Pavón-Carrasco[1,3], G. Cianchini[1], L. Perrone[1], C. Abbattista[4], L. Alfonsi[1], L. Amoruso[4], S. A. Campuzano[1], M. Carbone[4], C. Cesaroni[1], G. De Franceschi[1], Anna De Santis[1], R. Di Giovambattista[1], A. Ippolito[1,5], A. Piscini[1], D. Sabbagh[1], M. Soldani[1], F. Santoro[4], L. Spogli[1,6], R. Haagmans[7]

(1) Istituto Nazionale di Geofisica e Vulcanologia, Via di Vigna Murata 605, Rome 00143, Italy

(2) Now at School of Remote Sensing and Geomatics Engineering NUIST - Nanjing University of Information Science and Technology, Nanjing, China

(3) Now at Univ. Complutense de Madrid, Facultad CC. Físicas, Avd. Complutense, s/n – Madrid 28040, Spain & Geoscience Institute IGEO (CSIC – UCM), Madrid 28040, Spain.

(4) Planetek Italia srl, via Massaua 12, Bari 70132, Italy

(5) Now at ASI, Via del Politecnico snc, Roma 00133, Italy

(6) SpacEarth Technology, Via di Vigna Murata 605, Rome 00143, Italy

(7) European Space Agency, ESTEC, Keplerlaan 1, NL-2201, Noordwijk, The Netherlands

Captions of Figures S1-S3 and Table S1

Figure S1. Example of *NeAD* graphical output for a satellite *Swarm*-C track two days after the M7.8 Nepal EQ occurred on 25 April 2015. From left to right: the time derivative of Ne (dNe/dt, approximated by the first differences of Ne divided by the Ne sampling time, i.e. 0.5 s), residual of Ne with respect to cubic splines, geographical map with satellite track (red), Dobrovolsky area (yellow oval; circular on the terrestrial sphere) and epicentre (green star). The heading is self-explanatory: SAT=satellite A/B or C; Y/M/D= Date; Track Number; D=Down= Descending Orbit; LT=Local Time; UTC= Coordinated Universal Time; DST and AP are the Dst and a_p magnetic indices, respectively.

Figure S2. An example of output graph of *MASS* algorithm along a night-time track in the first differences of the X,Y,Z magnetic components and total intensity F (Satellite *Swarm* C) on 14 February 2016 (anomaly better visible in Y), i.e. almost two weeks before the M7.8 Sumatra earthquake occurred on 2 March 2016. The map on the right shows the area of interest (Dobrovolsky area, yellow circle; see its definition in the main text) and the orbital track for *Swarm* C satellite (red line). The star represents the Sumatra earthquake epicenter. The heading is self-explanatory: SAT=*Swarm* satellite A/B or C; Y/M/D= Date; Track Number; U=UP=Ascending Orbit; LT=Local Time; UTC= Coordinated Universal Time; Dst and ap are the Dst and a_p geomagnetic indices, respectively.

Figure S3. Simplified flowchart of the Worldwide Statistical Correlation (WSC) algorithm.

SAT.: C, Y/M/D: 2015/4/27, Track n:30 D, meanLT:3:54:24, meanUTC:22:43:8, DST=-21, Ap=6

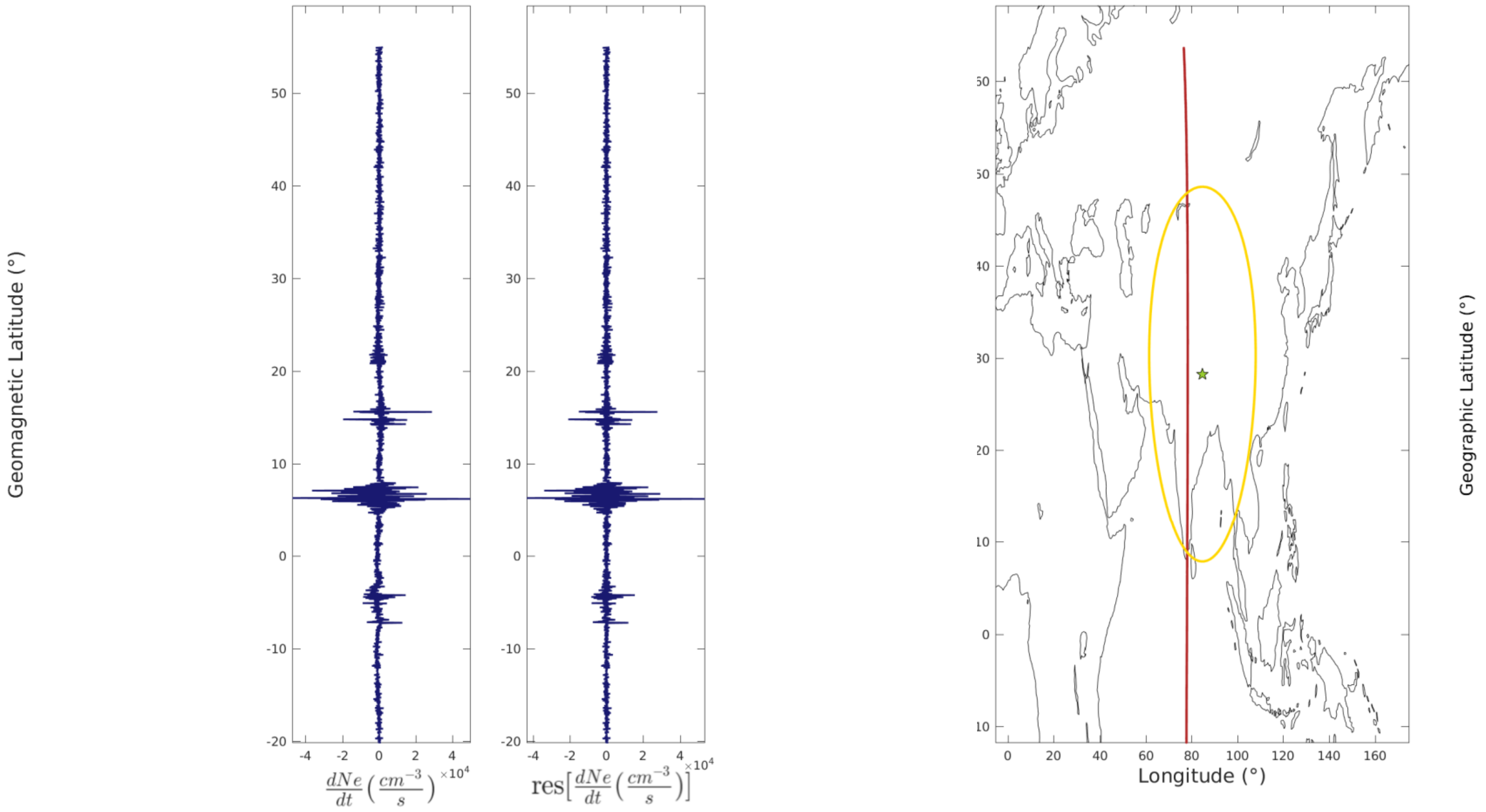

Figure S1

SAT.:C.Y/M/D:2016/2/14. Track n.:26 U.meanLT:1:42:27,meanUTC:18:16:53, Dst=-7nT, ap=6nT

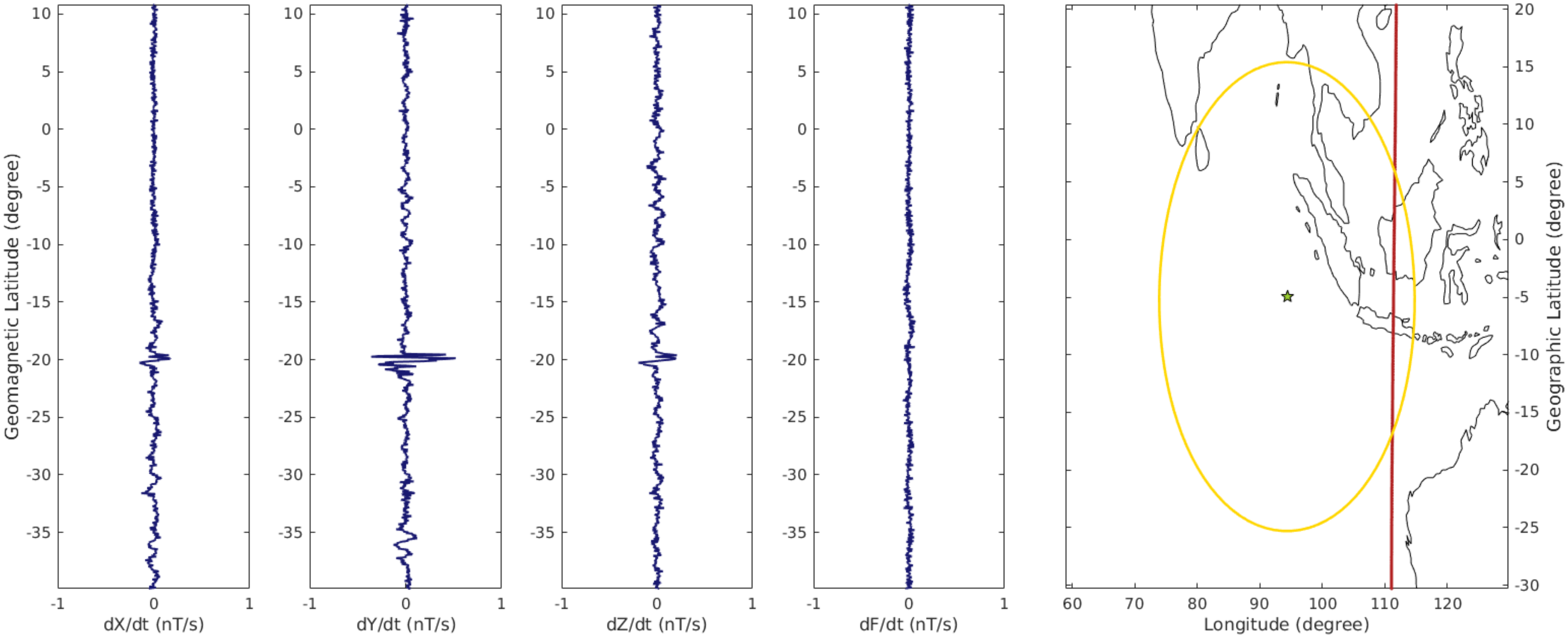

Figure S2

WSC between Swarm anomalies and Earthquakes

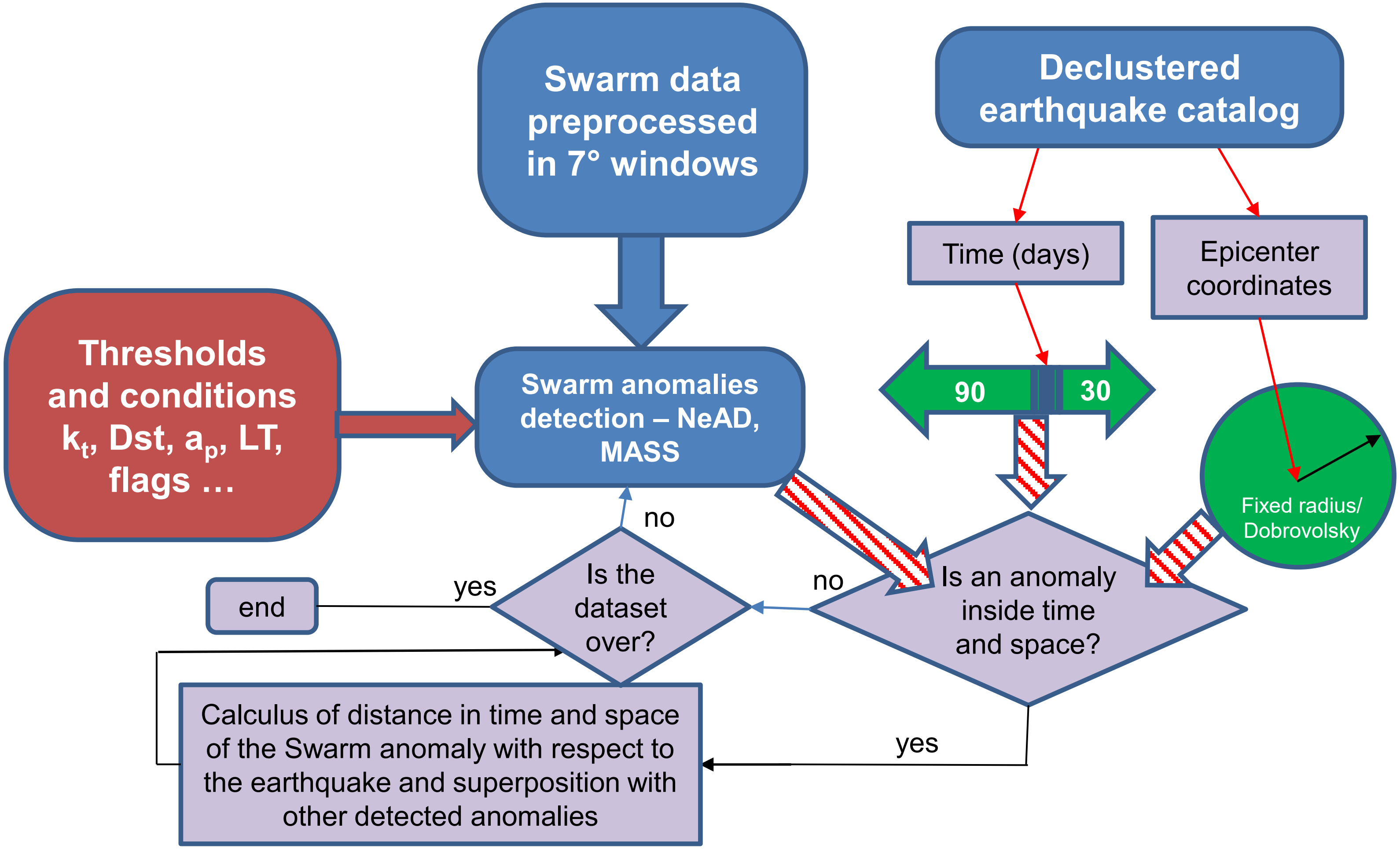

Figure S3

Table S1

	Anomalies in the whole space-time window	Anomalies in the 120 day window	EQs with anomalies	Anomalies in the max	EQs in the max	*[Dmax/D_0]$_{rand}$* $\pm\sigma_{rand}$
(Ne) 1000km Method 1	58692	37308	1242	129±4	121±3	1.10±0.02
(Ne) 1000km Method 2 - Log ΔT*R	58692	16347	1220	111±8	105±7	0.97±0.07
(Ne) 1000km Method 3 - MaxM	58692	16376	1057	59±3	56±3	0.58±0.04
(Ne) DbA Method 1	58692	9258	1084	93±6	88±7	0.91±0.05
(Ne) DbA Method 2 - Log ΔT*R	58692	6817	961	81±9	77±8	0.89±0.10
(Ne) DbA Method 3 - MaxM	58692	6817	754	57±2	54±2	0.80±0.04
(YMag) 1000km Method 1	22142	13896	1234	56±4	55±4	1.28±0.07
(YMag) 1000km Method 2 - Log ΔT*R	22142	6166	1132	45±5	44±5	1.10±0.11
(YMag) 1000km Method 3 - MaxM	22142	6166	943	27±2	25±2	0.76±0.07
(YMag) DbA Method 1	22142	3545	815	41±3	40±3	1.39±0.08
(YMag) DbA Method 2 - Log ΔT*R	22142	2567	663	33±3	32±3	1.38±0.12
(YMag) DbA Method 3 - MaxM	22142	2567	534	26±2	25±2	1.32±0.10

Table S1. Statistics for 100 random datasets of the cases analysed in the paper in all space-time interval compared with the values of the real data analyses. Ne/YMag at the lefter column are refered to the real analysis. [Dmax/D_0]$_{rand}$ is used to estimate *d* and *n* values given in Table 1. For each series of 100 random simulations we provide also the standard deviation σ_{rand}.
